# Supplementary material for: Plasticity in Gene Expression Patterns and CYPSF Gene Possibly Involved in the Etofenprox-Resistant Population of White-Backed Planthopper, Sogatella furcifera
Source: Int J Mol Sci. 2024 Dec 19;25(24):13605. doi: 10.3390/ijms252413605 (PMC11677642; doi:10.3390/ijms252413605)
Supplement: Supplementary file 1 [file ijms-25-13605-s001.zip › ijms-3320915-supplementary.pdf]

Table S1. Used primer lists

Primers	Sequence (5'->3')
WBPH_VSSC4S6_F	TCTCCTACCTGGTCATCAGCTT
WBPH_VSSC4S6_F1	CCTGGTCATCAGCTTCCTAATTGT
WBPH_VSSC4S6_R	GGACGTGGCCTTGCTCAAAC
WBPH_VSSC4S6_R1	TTAAGTGACTGACCTCGAGTGG
WBPH_VSSC4S6_R_nR1	ACGTGGCCTTGCTCAAACAT
WBPH_VSSC4S6_R_nR2	GCCAGTTTGGATGTCTGGGAA
WBPH_VSSC4S6_R_nR3	CTTGCGCGCGAAAAAGTCCTT
WBPH_VSSC_V253F-F	GGTTTGAAGACTATCGTCGGCG
WBPH_VSSC_V253F-R	TGTCATTGCTCATCCATCGGTG
WBPH_VSSC_M918T-F	CTGAGAGTGTTCAAACTGGCTA
WBPH_VSSC_M918T-R	TCGACATAGTTCTTCCCAAATAGC
WBPH_VSSC_L1014F-F	CGCTGGAACTTCACCGATTTCA
WBPH_VSSC_L1014F-R	GAGCCGAAATTCGACAGCAACA
WBPH_VSSC_F1534C-F	CAAACAACCTATCAGGGAGACG
WBPH_VSSC_F1534C-R	GCTCGTTGAAATTGTCAATGATTACTCC
WBPH_VSSC_F1534C-Fin-1	CAGTCCACAGTGTTCGTGACTT
WBPH_VSSC_F1534C-Fin-2	ACCGAGAGACCAATTGCCAGTA

Table S2. Raw data stats

Index	Sample id	Total read bases*	Total reads	GC (%)	Q30 (%)
1	JD_100R-1	13,500,039,760	133,663,760	44.12	95.34
2	JD_100R-2	12,350,318,178	122,280,378	42.96	95.33
3	JD_100R-3	14,597,504,346	144,529,746	43.52	95.09
4	JD_25R-1	13,388,511,116	132,559,516	42.69	95.43
5	JD_25R-2	12,218,586,302	120,976,102	41.88	95.26
6	JD_25R-3	11,349,882,474	112,375,074	42.46	95.33
7	JD_NT-1	11,380,951,690	112,682,690	42.84	95.50
8	JD_NT-2	11,572,504,654	114,579,254	43.17	95.27
9	JD_NT-3	12,215,710,024	120,947,624	42.73	95.49

· Total read bases: Total number of read bases after trimming
· Total reads: Total number of reads after trimming
· GC (%): GC Content
· Q30 (%): Ratio of bases that have phred quality score greater than or equal to 30

Table S3. Trimming Data Stats

Index	Sample id	Total read bases*	Total reads	GC (%)	Q30 (%)
1	JD_100R-1	13,259,957,947	131,877,316	44.12	95.93
2	JD_100R-2	12,126,704,352	120,609,126	42.96	95.93
3	JD_100R-3	14,325,306,085	142,491,008	43.52	95.71
4	JD_25R-1	13,153,742,294	130,858,700	42.69	95.99
5	JD_25R-2	11,985,580,709	119,257,840	41.90	95.88
6	JD_25R-3	11,142,861,357	110,856,998	42.45	95.92
7	JD_NT-1	11,187,754,488	111,260,112	42.83	96.08
8	JD_NT-2	11,355,057,946	112,931,972	43.17	95.91
9	JD_NT-3	11,996,716,911	119,435,754	42.72	96.04

· Total read bases: Total number of read bases after trimming
· Total reads: Total number of reads after trimming
· GC (%): GC Content
· Q30 (%): Ratio of bases that have phred quality score greater than or equal to 30

Table S4. Overall mapping ratio in the reference unigene set (merged) for each sample

Sample	Number of processed reads	Number of mapped reads	Number of unmapped reads
JD_100R-1	131,877,316	105,239,130 (79.8%)	26,638,186 (20.2%)
JD_100R-2	120,609,126	94,801,342 (78.6%)	25,807,784 (21.4%)
JD_100R-3	142,491,008	115,055,930 (80.75%)	27,435,078 (19.25%)
JD_25R-1	130,858,700	103,096,944 (78.78%)	27,761,756 (21.22%)
JD_25R-2	119,257,840	95,004,554 (79.66%)	24,253,286 (20.34%)
JD_25R-3	110,856,998	88,167,586 (79.53%)	22,689,412 (20.47%)
JD_NT-1	111,260,112	88,280,048 (79.35%)	22,980,064 (20.65%)
JD_NT-2	112,931,972	90,591,368 (80.22%)	22,340,604 (19.78%)
JD_NT-3	119,435,754	92,586,808 (77.52%)	26,848,946 (22.48%)
